# A Case Report of Bowel Perforation in a Gravid COVID-19 Patient

**DOI:** 10.1155/2023/7737433

**Published:** 2023-03-08

**Authors:** A. T. Guiritan, J. G. Cataluña

**Affiliations:** St. Luke's Medical Center, Quezon City, Philippines

## Abstract

**Background:**

Bowel perforation in a pregnant patient with COVID-19 infection is a rarely reported complication. With the uncovering of the different treatment modalities and complications of this disease, this case highlights diagnostic and therapeutic dilemmas and limitations in a special population. *Clinical Presentation*. A 35-year-old female, Gravida 2 Para 1 at 31 weeks age of gestation (AOG) who underwent cesarean section for nonreassuring fetal status in 2019, was admitted for severe COVID-19 infection presenting with dyspnea. During her hospitalization, she developed progressive dyspnea from worsening of COVID-19 infection. Patient was eventually intubated and was given a total of two doses of tocilizumab, adequate antibiotic treatment, remdesivir, and dexamethasone. An emergency repeat cesarean section was performed due to maternal deterioration and poor variability of the fetus. She delivered to a live preterm baby girl, with an Apgar score of 1 and 7 on the 1st and 5th minute of life respectively. During the postoperative days, patient remained intubated and sedated. Increasing residuals were noted per nasogastric tube (NGT). Serial scout film of the abdomen (SFA) and medical management were performed. Upon weaning from sedation, patient complained of right lower quadrant pain. A whole abdominal computed tomography (CT) scan with contrast was requested which revealed large bowel obstruction. Patient was referred to surgery service. Upon assessment, abdomen was noted to be rigid with guarding during palpation. A scout film of the abdomen was requested and revealed extensive amount of pneumoperitoneum with scanty to absent colonic gas suggestive of bowel perforation. Patient underwent emergency exploratory laparotomy. Upon opening the peritoneum, free air was evacuated. Approximately 1.4 liters of fecaloid peritoneal fluid was suctioned and adhesive band was noted at the pelvic area. A 2 cm full thickness perforation at the cecum with 17 cm serosal tear from the cecum to ascending colon was noted. Bowel loops proximal to the perforation were dilated. Nasogastric tube was inserted for bowel decompression. Right hemicolectomy was performed. Specimen was sent for histopathology. Acute inflammatory cells infiltrating the blood vessels and possible microthrombi and beginning thrombus formation were noted in the subserosa. Patient eventually expired on the 14^th^ hospital day from sepsis.

**Conclusion:**

This case highlights a rarely reported complication of COVID-19 infection. Confluence of factors that predisposed the patient include pregnancy, COVID-19 infection, use of tocilizumab, and recent surgery. High index of suspicion is vital in the management and improvement of outcomes.

## 1. Introduction

Coronavirus disease, caused by severe acute respiratory syndrome coronavirus 2 (SARS-COV-2), was initially characterized with respiratory symptoms. However, patients with severe to critical COVID-19 infection are at a particularly high risk for developing gastrointestinal complications due to prolonged hospitalization [1].

Pregnant people may be particularly susceptible to COVID-19 because the physiologic changes of pregnancy involve alterations in immune system, which may result in an altered response to infection during pregnancy [2]. Morelli et al. in 1953 were the first to propose the concept of immune tolerance during pregnancy [3]. They hypothesized that the semi allogeneic fetus is able to survive due to regulation of the immunologic interactions between the mother and the fetus. Such regulation can be caused by a lack of fetal antigen expression and/or functional suppression of maternal immune response which could possibly predispose a pregnant patient to severe COVID-19 infection.

The conception of different therapeutic modalities for this disease has been evolving. The COVID-19 treatment guidelines panel recommends against withholding treatment for COVID-19 infection in a pregnant patient [4]. Termination of pregnancy and mode of delivery are also important issues in this population. Cesarean delivery should not be considered medically reasonable until it becomes, ethically or medically obligatory for either maternal or fetal indications [5].

This is a case of bowel perforation in a 35-year-old female patient with critical COVID-19 infection with no past history of gastrointestinal disease.

## 2. Case Presentation

A 35-year-old female, Gravida 2 Para 1 at 31 weeks age of gestation (AOG) who underwent cesarean section for nonreassuring fetal status in 2019, coming in for dyspnea. She had no history of gastrointestinal disease and had no COVID-19 vaccination.

At the time of admission, the patient was conscious, coherent, and in mild cardiorespiratory distress and with desaturation as low as 88% at room air. Examination of the chest revealed bibasal crackles. The abdomen was globular, normoactive, with palpable good fetal movement and good fetal heart tones were appreciated, and was nontender on all quadrants. She was initially managed as a case of severe COVID-19 infection and was given adequate antibiotics, a total of two doses of tocilizumab, remdesivir, and dexamethasone.

**Figure 1:**
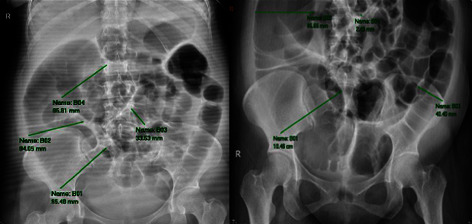
Serial scout film of the abdomen. Description: regression of ileus; there is a decrease in number and amount of air-filled small bowel segments with no organizing pattern.

During her admission, patient had progression of dyspnea and hemodynamic instability, hence intubation was performed. It was also noted that the fetus had poor variability in the fetal cardiotocogram. A decision was made to perform an emergency repeat cesarean section. She delivered to a live preterm baby girl, with an Apgar score of 1 and 7 on the 1st and 5th minute of life respectively. Apgar score 1, 7 with a birth weight of 1520 grams. After the first week of admission, residuals of 150 milliliters per nasogastric tube (NGT) with no bowel movement for 3 days was noted. Medical management was conducted. Serial scout film of the abdomen (SFA) revealed only the presence of ileus with no evidence of pneumoperitoneum (Figure 1).

**Figure 2:**
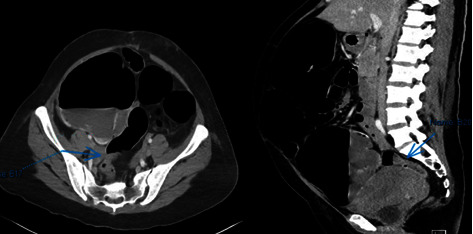
Whole abdominal CT scan with contrast. Description: large bowel obstruction with transition point at the rectosigmoid junction, likely from postoperative adhesions; pneumatosis coli in the cecum and ascending colon; no ascites, pneumoperitoneum, or enlarged lymph nodes; prominent-sized appendix without inflammatory changes; and anteverted and globularly enlarged uterus with a prominent endometrium.

While weaning from sedation, she complained of right lower quadrant pain. On assessment, abdomen was soft but with direct tenderness at the right lower quadrant with consideration of possible appendicitis. Hence, A whole abdominal computed tomography (CT) scan with contrast (Figure 2) was requested which revealed large bowel obstruction. Patient was then referred to surgery service.

**Figure 3:**
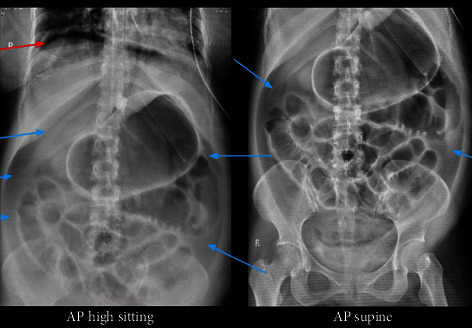
Scout film of the abdomen. Follow-up study to the previous scout film of the abdomen, now demonstrates extensive amount of free intraperitoneal air as pointed by the blue arrows with lucency in the subdiaphragmatic area as pointed by the red arrow. Description: interval demonstration of extensive amount of pneumoperitoneum with scanty to absent colonic gas in the present study; regression of the ileus; gastric distention.

On assessment, abdomen was noted to be rigid with guarding during palpation. A scout film of the abdomen (Figure 3) was requested and revealed extensive amount of free intraperitoneal air (as pointed by the blue arrows) with lucency in the subdiaphragmatic area (as pointed by the red arrow). The patient underwent emergency exploratory laparotomy.

**Figure 4:**
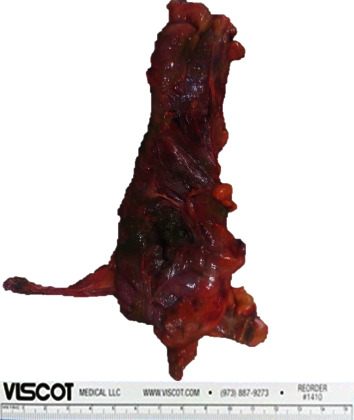
Gross specimen. Description: upon opening the peritoneum, free air was evacuated. Approximately 1.4 L of fecaloid peritoneal fluid was suctioned. Adhesive band noted at the pelvic area. Noted a 2 cm full thickness perforation at the cecum. 17 cm serosal tear from the cecum to ascending colon. Bowel loops proximal to the perforation were dilated.

Upon opening the peritoneum, free air was evacuated. Approximately 1.4 liters of fecaloid peritoneal fluid was suctioned and adhesive band was noted at the pelvic area. A 2 cm full thickness perforation at the cecum with 17 cm serosal tear from the cecum to ascending colon was noted. Bowel loops proximal to the perforation were dilated. Bowel decompression was performed via NGT. Right hemicolectomy was performed. Specimen (terminal ileum to proximal transverse colon) was sent for histopathology (Figure 4) which revealed acute inflammatory cells infiltrating the blood vessels and possible microthrombi and beginning thrombus formation were noted in the subserosa. Patient eventually expired on the 14th hospital day from sepsis.

## 3. Discussion

**Figure 5:**
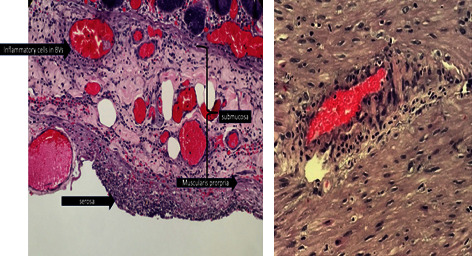
Blood vessel infiltrated with acute inflammatory cells and areas in the subserosa with possible microthrombi and beginning thrombus formation.

**Figure 6:**
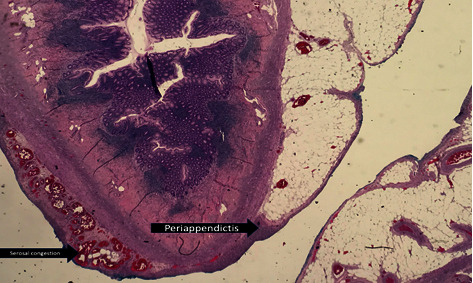
Serosal congestion and periappendicitis.

Pregnant women do not appear to have an increased risk of acquiring COVID-19 infection. However, those who are infected (Figure 5) seem to be at higher risk (Figure 6) for developing severe illness [2]. Majority of symptomatic gravid COVID-19 patients were at their third trimester of pregnancy [6]. In a study conducted by Allotey et al., it was reported that the prevalence of all-cause mortality was 0.63%, severe COVID-19 was 13%, admission to an intensive care unit (ICU) was 4%, and pregnant women requiring invasive ventilation was at 3% [7]. For the fetal considerations, impact of prolonged maternal hypoxemia, which may be significant, given the protracted clinical course of COVID-19, balanced against the risks associated with delivery at a given gestational age. The fetal risks associated with prematurity are well known and decrease with increasing gestational age at delivery [5]. Failure to perform cesarean delivery when this deliberative clinical judgment is justified increases risk of harm, bothto the fetus and the pregnant patient. Hence, ethically speaking, the mother may be allowed to subject herself and the fetus to a reasonable risk for their mutual benefit.

Although adhesions from previous abdominal surgery could have predisposed the patient to developing bowel obstruction leading to subsequent bowel perforation, we explored other contributory factors that led to this complication.

Throughout pandemic it appears that patients with COVID-19 fulfill the classic Virchow's triad required for development of thrombosis [8]. Endothelial injury associated with immune complex-mediated vasculitis has also been postulated as one of the mechanisms behind vascular damage in COVID-19. Both of these in combination can cause endothelial dysfunction and predispose a patient to thrombus formation. Pregnancy is a hypercoagulable state with increased thrombin production and increase in intravascular inflammation. During pregnancy, there are higher levels of circulating coagulation and fibrinolytic factors, such as plasmin, and these may be implicated in the pathogenesis of SARS-CoV-2 infection. Pregnant women are at increased risk of thromboembolic events with associated mortality. Therefore, pregnant women with COVID-19 may have additive or synergistic risk factors for thrombosis. Stasis can be expected in all critically ill patients because of isolation in a confined area, prolonged bed rest, immobilization in the intensive care unit, and possible limitations to physiotherapy. Lastly, although not part of Virchow's triad, hemodynamic instability in severe COVID-19 infection leading to hypotension and shock can be a possible mechanism of nonocclusive mesenteric ischemia seen in these patients.

Another possible but rare etiology for bowel perforation is the use of tocilizumab infusion. Tocilizumab is an interleukin-6 receptor antagonist that is being used for the profound inflammatory state associated with COVID-19 infection. In mice, interleukin-6 stimulates intestinal epithelial proliferation, decreases intestinal injury, and improves barrier function following ischemia reperfusion of the small bowel. Interleukin-6 null mice exhibited impaired recovery following massive enterectomy and increased apoptosis [9]. Therefore, tocilizumab may interfere with recovery from intestinal injuries caused by diverticulitis or other gastrointestinal conditions.

Pregnant women with COVID-19 infection had worse consequences compared with nonpregnant women [10]. In critically ill patients with COVID-19, prognosis is poor with mortality ranging from 12 to 78% with a mean average of 25 to 50 percent, and 92% of the deaths occurring within the immediate postoperative days due to multiorgan failure or refractory septic shock [11].

Our patient was pregnant with COVID-19 infection. The modulations of the maternal immune system in pregnancy may affect the response to infections, and specifically to viruses. The altered inflammatory response to viruses during pregnancy is thought to be mediated, at least in part, by the certain mechanisms which can predispose the patient to severe to critical COVID-19 infection in pregnancy necessitating an emergency repeat cesarean section delivery because of deteriorating maternal status which predisposed her to developing adhesions. These adhesions could have predisposed the patient to developing large bowel obstruction and subsequent bowel perforation. Pregnancy placed the patient in a hypercoagulable state that may lead to formation of microthrombi causing ileus. Other factor such as recent surgery could also lead to ileus, which again could lead to large bowel obstruction and subsequent bowel perforation. In severe to critical COVID-19, tocilizumab, an interleukin-6 antagonist, is used on compassionate grounds for treatment of COVID-19 cytokine storm [12]. In a study conducted by Nakajima et al., they noted that there is no increased rates of spontaneous abortion or congenital abnormalities secondary to exposure to tocilizumab during pregnancy [13]. However, tocilizumab may interfere with recovery from injuries due to impaired intestinal cell proliferation leading to bowel ulceration. Gastrointestinal (GI) perforation is an uncommon but serious adverse effect in tocilizumab therapy.

## 4. Conclusion

The modulations of the maternal immune system in pregnancy may affect the response to infections, and specifically to viruses. Pregnant women do not appear to have an increased risk of acquiring COVID-19 infection. However, those who are infected seem to be at higher risk for developing severe illness. Patients with severe COVID-19 are at a particularly high risk for developing gastrointestinal complications. Gastrointestinal perforation is an uncommon but serious adverse event in tocilizumab therapy and COVID-19 related vasculitis. High index of suspicion is vital in the management and improvement of outcomes.

## Data Availability

The article is a case report written regarding a gravid patient who developed COVID-19 and its complications. The data can be accessed upon request.
